# Cognitive Fatigue Disrupts Explosive Performance and Vigilance in Trained Individuals

**DOI:** 10.3390/sports13110386

**Published:** 2025-11-04

**Authors:** Andreas Stafylidis, Walter Staiano, Athanasios Mandroukas, Yiannis Michailidis, Lluis Raimon Salazar Bonet, Angelos E. Kyranoudis, Marco Romagnoli, Ana Ferri-Caruana, Thomas I. Metaxas

**Affiliations:** 1 Laboratory of Evaluation of Human Biological Performance, Department of Physical Education and Sports Sciences, Aristotle University of Thessaloniki, University Campus of Thermi, 57001 Thessaloniki, Greece; astafylidis@phed.auth.gr (A.S.); amandrou@phed.auth.gr (A.M.); ioannimd@phed.auth.gr (Y.M.); aggelos1970@gmail.com (A.E.K.); tommet@phed.auth.gr (T.I.M.); 2 Department of Physical Education and Sport, University of Valencia, 46000 Valencia, Spain; marco.romagnoli@uv.es (M.R.); ana.maria.ferri@uv.es (A.F.-C.); 3 International University SEK, Quito 170151, Ecuador; raimon.salazar@uisek.edu.ec

**Keywords:** mental fatigue, repeated sprint ability, psychomotor vigilance, subjective workload, repeated jumping ability, reaction time, cognitive load

## Abstract

This study examined the effects of cognitive fatigue on repeated sprint ability (RSA), neuromuscular performance, and vigilance in physically active young adults (N = 28, 16 males, 12 females; mean age = 20.6 ± 1.4 years). Participants were randomly assigned to a mental fatigue (MF) or control (CON) group and completed baseline and post-condition assessments following a cognitively demanding or neutral task. Repeated sprint performance significantly declined in the MF group, as shown by increased RSA mean time (Δ = 0.432 s, *p* < 0.001) and total time (Δ = 4.331 s, *p* < 0.001), with no statistically significant change observed in the CON group. Countermovement jump height remained unaffected; however, repeated jump ability showed impaired contact time (Δ = 0.084 s, *p* = 0.007) in the MF group. Psychomotor vigilance significantly deteriorated under mental fatigue, as evidenced by slower reaction times (Δ = 119.71 ms, *p* < 0.001) and increased lapses (Δ = 2.86, *p* < 0.001). Subjective ratings confirmed elevated perceived exertion (Δ = 0.79, *p* = 0.002) and mental fatigue (Δ = 8.00, *p* < 0.001) in the MF group, without changes in motivation. These findings may demonstrate that cognitive fatigue impairs both physical and cognitive performance, even in trained individuals.

## 1. Introduction

Mental fatigue (MF) is a psychobiological state characterized by subjective sensations of tiredness or diminished energy and impaired cognitive functioning, typically induced by cognitively demanding activities [1,2]. Research in sports science has established that MF can negatively influence aerobic endurance performance [1,3] and sport-specific psychomotor performance, complex behaviors requiring the integration of cognitive processing and perceptual information within sporting contexts [4].

In contrast, the influence of MF on anaerobic output and neuromuscular performance (e.g., maximal force and power production involving the muscular and nervous systems) has received comparatively less attention. Systematic reviews indicate that MF does not appear to affect short, maximal bursts of activity, such as sprints, jumps, or maximal strength tasks [1,5], with several studies showing no impairment in squat jump (SJ) [6] countermovement jump (CMJ) [7,8,9] and sprinting performance due to MF [8]. Similarly, other investigations have reported no detrimental effects on brief strength or power activities and sprint performance lasting less than three minutes [6,10,11]. For example, prior work found that linear repeated sprint ability (RSA) was unaffected by MF [9]. Yet, a separate line of evidence on strength endurance has found that MF can reduce performance in submaximal endurance tasks [8,9,10], such as repeated submaximal contractions, where MF increases time to exhaustion [12] or decreases the number of repetitions and sets in resistance training [7,13]. This suggests that, like aerobic endurance, strength endurance may be particularly susceptible to MF, potentially due to heightened cognitive involvement in pacing, decision making, and moderating perceived effort [5,14].

Moreover, these cognitive demands are amplified in sporting environments where athletes must interpret situational information and adjust movements, such as balance, postural control, and reactive stabilization, while facing complex physical demands [15,16,17,18]. In such multifaceted contexts, MF may further impair decision making, technical skills, and adaptability. However, research examining MF’s impact on anaerobic and strength endurance performance remains limited to studies employing isolated physical tests without considering the full spectrum of cognitive elements inherent to sport. For greater ecological validity, experimental protocols should examine the interaction of MF and physical fatigue during tasks integrating posture, balance, rapid decision making, and visuo-motor reactions [19,20,21].

Existing studies using repeated jump ability (RJA) protocols have primarily focused on environmental stressors, such as heat, rather than MF [22]. Similarly, research on RSA has largely been confined to linear sprint assessments. To date, the only study that has investigated the combined effects of mental fatigue on both RSA (incorporating linear and directional components) and RJA is the recent work by Staiano et al. [9]. Their findings demonstrated that a mentally fatiguing task can impair repeated jump and sprint performance in team sport athletes, providing the first experimental evidence in this specific area.

Given the novelty and importance of these findings and limited research, the present study was designed as a replication of Staiano et al. [9]. Our aim was to systematically investigate whether MF, induced by a demanding cognitive task, would similarly affect repeated sprint ability (RSA) and repeated jump ability (RJA), as well as vigilance (i.e., sustained attention over time), in a new cohort. Replicating this protocol is crucial to confirm and extend the generalizability of the initial results, as Staiano et al.’s study [9] remains the only available reference on this precise topic to date.

We hypothesized that MF would impair performance on this multidirectional RSA, increase perceived exertion, and reduce mean performance in RJA, while not affecting peak outputs in isolated tasks. Additionally, we anticipated that MF would further impair psychomotor vigilance (as measured by sustained attention and reaction time), particularly when combined with physical fatigue.

## 2. Materials and Methods

### 2.1. Participants

Twenty-eight physically active university students (16 males and 12 females; mean age males: 20.6 ± 1.15 years; females: 20.7 ± 1.67 years) volunteered for the study. Participants were recruited from the Department of Physical Education and Sport Science and were amateur athletes training at least three times per week for 5 years (level 2 trained) [23]. Anthropometric data showed males averaged 178.7 ± 9.04 cm in height and 75.7 ± 11.38 kg in weight, while females averaged 166.9 ± 5.23 cm and 59.6 ± 4.03 kg, respectively. Ethical approval was obtained from the University of Thessaloniki (Approval No. 203/2024), and all participants provided written informed consent.

### 2.2. Design

**Figure 1:**
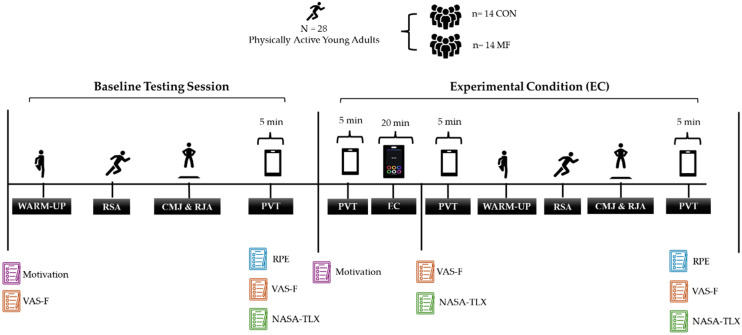
Schematic representation of the experimental procedure. Note: The figure provides an overview of the assessment sequence and experimental procedures implemented for the mental fatigue (MF) and control (CON) groups. The protocol included a standardized warm-up, followed by baseline measurements of repeated sprint ability (RSA), countermovement jump (CMJ), repeated jumping ability (RJA), rating of perceived exertion (RPE), and the Psychomotor Vigilance Test (PVT). Participants were then exposed to the experimental condition (EC), consisting of a cognitively demanding Stroop task for the MF group or a non-fatiguing control activity for the CON group. Post-intervention assessments (2nd Battery) were conducted using the same battery of physical and cognitive tests. Abbreviations: RSA = repeated sprint ability; CMJ = countermovement jump; RJA = repeated jumping ability; PVT = Psychomotor Vigilance Test; EC = experimental condition; CON = control group; MF = mental fatigue group.

The study followed a randomized, between-group experimental design comprising a familiarization session and one testing session, separated by at least 10 days to minimize residual fatigue (Figure 1). Participants were randomly assigned (stratified by gender) to either the MF or CON group. Inclusion criteria were being in good health, no injuries in the previous six months, and prior experience with CMJ assessments. Two blinded independent investigators managed the assessments; one conducted the MF intervention, and the other, blinded to group allocation, performed physical and cognitive testing. Participants were instructed to maintain proper hydration and sleep quality and refrain from caffeine, alcohol, and vigorous exercise for 48 h before testing. All testing was conducted under controlled and similar environmental conditions. Participants were unaware of the aim of the study, and they were debriefed upon completion.

### 2.3. Procedures

During the familiarization session, demographic and anthropometric data were collected, and participants were introduced to all testing protocols, including the CMJ [24,25], RSA [26,27], and RJA tests [28]. Additionally, participants were familiarized with the Stroop task [29], the Psychomotor Vigilance Test (PVT) [30] using the SOMA-NPT app (Soma Technologies, Lucerne, Switzerland), which has been used in numerous peer-reviewed studies [9,31,32,33], the NASA Task Load Index (NASA-TLX) [34], the visual analog scale for mental fatigue (M-VAS) [6,9], and the Borg CR-10 Rating of Perceived Exertion (RPE) scale [35].

During the testing session, participants rated their motivation to complete the whole testing session, completed the M-VAS for mental fatigue, engaged in a standardized warm-up (15 min, including running at a low-to-moderate intensity and dynamic stretching exercises), and performed the RSA test. The multi-directional RSA test protocol involved ten 30 m sprints with multiple changes of direction (6 × 5 m) and 30 s of passive recovery between each sprint, following procedures used in prior studies [26,27]. At completion, they rated their RPE. For the RSA analysis, performance metrics were defined as follows: the best time (BT) referred to the fastest sprint recorded, the mean time the average of all 10 sprints, and the total time (TT) the cumulative duration of all sprints. The coefficient of variation (CV, %) was calculated as the ratio of the standard deviation to the mean, and the decrement score (Sdec) [36,37] was also analyzed. The percentage decrement score (Sdec) was utilized as a reliable and valid method for quantifying fatigue during RSA tests [36,37]. Sprint times were measured with an accuracy of 0.01 s using infrared photoelectric gates (Microgate, Bolzano, Italy), consistent with prior research methodologies [38]. Afterwards, participants were provided with clear instructions on performing the CMJ without using an arm swing. During the procedure, participants were required to maintain a standing position with their hands placed on their hips, perform a countermovement to a self-selected depth, and jump as high as possible when ready. Each participant performed three maximal-effort CMJs, with a two-minute recovery period between attempts. For analysis, the highest recorded jump was selected, aligning with methods employed in previous research [24,25,28]. Jump heights were recorded using a smartphone and subsequently analyzed using the My Jump 2 application. Afterwards, upon hearing a verbal cue, they performed repeated vertical jumps for 15 s, aiming for maximum height while ensuring simultaneous landing with both feet after each jump. Participants were instructed to “jump as high as possible during the 15-s maximal jump test,” following the protocol established by Del Coso et al. [39]. All participants were carefully supervised to ensure identical posture and execution, including hands placed on the hips, upright trunk alignment, and avoidance of arm swing, thereby maintaining consistency across trials. The test employed a CMJ technique without the use of upper limbs, as outlined by Hespanhol et al. [40]. Participants performed each jump with an approximate knee flexion angle of 110°, which has been identified as optimal for maximizing strength application [40,41]. Continuous vertical jumps were performed at maximal effort, with no pauses between repetitions. To ensure accuracy and consistency, participants were instructed to maintain a vertical trunk position, minimize forward motion, and fully extend their knees during the flight phase of each jump. The average vertical jump height over the 15 s interval was calculated, in line with methods validated in prior studies [39,40]. At completion, they rated their RPE and completed the NASA TLX scales and M-VAS. Afterwards, they performed a 5 min PVT using SOMA NPT (Lucerne, Switzerland) to assess vigilance to perform. Next, they completed one of two 30 min experimental conditions while sitting comfortably in a quiet, dimly lit room. In the MF condition, they performed an incongruent Stroop color–word task using a tablet running the SOMA-NPT app (Switch, Lucerne, Switzerland). In the control condition (CON), they watched an emotionally neutral documentary about cars on the same screen [1]. At the end of each condition, they completed again the National Aeronautics and Space Administration Task Load Index (NASA-TLX), performed a 5 min PVT, and rated their MF. Afterwards, they repeated the battery of physical tests again (RSA, CMJ, RJA), filled out the questionnaire, and completed another 5 min PVT.

In this study, the My Jump 2 mobile application was operated using an iPhone 15 Pro (Apple Inc., Cupertino, CA, USA) equipped with a 240 Hz video recording capability, ensuring high precision in motion capture. The device was securely placed on a stable support at a distance of 1.5 m from the participant to maintain stability and minimize recording inaccuracies, as recommended by Bogataj et al. [42]. The accuracy and reliability of the My Jump application have been extensively validated in previous research [24,42,43].

### 2.4. Measures

Motivation to complete the testing session was assessed using a 5-point Likert scale. Participants rated their agreement with the statement “I am motivated to perform the test” on a scale ranging from 0 (not at all) to 4 (extremely). This method aligns with protocols used in prior research to quantify motivation levels during physical performance assessments [44]. The NASA-TLX was employed to measure subjective workload following established guidelines [34]. As in previous research [9], four subscales were utilized: mental demand (assessing the cognitive and perceptual activity required), physical demand (evaluating the physical exertion needed), effort (measuring the intensity of work required to achieve the performance level), and frustration (perceived levels of irritation or annoyance during the task). Participants rated each subscale on a scale divided into 20 equal intervals anchored by bipolar descriptors (e.g., high/low). These ratings were multiplied by 5, yielding a final score ranging from 0 to 100 for each subscale. A visual analog scale (VAS) was employed to assess subjective mental fatigue (MF) before and after each condition. Specifically, the Mental Visual Analog Scale (M-VAS) was used, on which participants marked their perceived level of MF on a 10 cm line visual analog scale with descriptors from “not at all exhausted” to “completely exhausted.” This measurement was conducted both before and after the cognitively demanding task and the natural condition (CON), following the procedure outlined by Smith et al. [45]. The rating of perceived exertion (RPE) was measured using the Borg CR-10 scale. Participants received detailed instructions on how to use the scale and were shown the RPE chart to ensure a clear understanding of what each number represented. After each sprint in the RSA test, following the RJA, and at the conclusion of all measurements, participants were asked “How was your workout?” and prompted to provide their response using the scale.

### 2.5. Statistical Analysis

All data are presented as mean ± standard deviation (SD), unless otherwise stated. Normality of the data was assessed using the Shapiro–Wilk test, complemented by visual inspection of histograms, Q–Q plots, and boxplots.

To determine the minimum required sample size for detecting a statistically significant group × time interaction, an a priori power analysis was performed using G*Power (Version 3.1.9.7) [46,47] in a 2 (group: MF vs. CON) × 2 (time: pre vs. post) mixed-design ANOVA. The power analysis was conducted using the “Repeated Measures: Within–Between Interaction” module, with parameters set to a large effect size (f = 0.40), an alpha level of 0.05, a statistical power of 0.80 (1 − β), and an assumed correlation of 0.50 between repeated measures. The nonsphericity correction (ε) was set at 1.0. The analysis indicated that a minimum of 16 participants (8 per group) would be required to detect a significant effect. Nevertheless, to enhance statistical power and account for potential data loss or interindividual variability, a larger sample was recruited, resulting in a total of 28 participants (14 per group). This decision aligns with methodological precedent in the field, such as the study by Queirós et al. [7], who used a within-subjects design with a comparable effect size.

The primary outcomes were analyzed using 2 × 2 mixed-design repeated-measures ANOVAs, incorporating time (pre vs. post) as the within-subjects factor and group (CON vs. MF) as the between-subjects factor. For specific dependent variables collected at three time points (e.g., NASA-TLX subscales, reaction time, and lapses), 2 × 3 mixed-design ANOVAs were conducted, with time defined as baseline, EC–30 min, and EC–2nd Battery. Where Mauchly’s test indicated a violation of the sphericity assumption, Greenhouse–Geisser corrections were applied. Bonferroni-adjusted pairwise comparisons were applied for post hoc analyses. For all pairwise comparisons, Cohen’s d was calculated to interpret the magnitude of differences, with 95% confidence intervals (CIs) provided where relevant.

The statistical analyses were conducted using SPSS (Version 29), Jamovi (Version 2.6) and JASP (version 0.19.3) [48,49,50]. Effect sizes (ESs) for ANOVA tests were expressed using partial eta squared (η^2^_p_) and interpreted in accordance with established thresholds, small (0.01 ≤ η^2^_p_ < 0.06), medium (0.06 ≤ η^2^_p_ < 0.14), and large (η^2^_p_ ≥ 0.14), following the guidelines of Cohen and Richardson [51,52]. For comparisons involving *t*-tests, Cohen’s *d* was used to estimate the magnitude of effects, categorized as small (d < 0.50), medium (0.50 ≤ d < 0.80), and large (d ≥ 0.80). CIs for Cohen’s *d* were set at 95%, reflecting the two-tailed nature of these tests and the possibility of both positive and negative values. In contrast, partial eta squared values were accompanied by 90% CIs, consistent with methodological recommendations for F-tests, which are typically one-sided and yield only non-negative estimates. This approach avoids interpretative ambiguity, as 95% CIs may include 0 despite statistical significance due to the bounded nature of squared effect size metrics [53,54,55]. CIs for η^2^_p_ were calculated using SPSS syntax scripts provided by Wuensch [54], based on the approach described by Smithson [56]. This methodological strategy ensures more accurate and interpretable effect size reporting in the context of ANOVA-based analyses [53,55]. The threshold for statistical significance was established at *p* < 0.05.

## 3. Results

### 3.1. Performance Measures—Repeated Sprint Ability (RSA)

#### 3.1.1. Best Performance

A significant time × group interaction was found, F(1, 26) = 6.19, *p* = 0.020, η^2^_p_ = 0.192, 90% CI [0.02, 0.45], indicating divergent responses from baseline to EC. However, no significant within-group changes were observed in either the MF group (Δ = −0.149 s, *p* = 0.953, d = −0.46, 95% CI [−1.39, 0.47]) or the CON group (Δ = 0.213 s, *p* = 0.292, d = 0.66, 95% CI [−0.29, 1.61]). The groups also did not differ at EC (Δ = −0.190 s, *p* = 1.000, d = −0.59, 95% CI [−1.94, 0.76]). Main effects of time, F(1, 26) = 0.19, *p* = 0.666, η^2^_p_ = 0.007, and group, F(1, 26) = 0.01, *p* = 0.928, η^2^_p_ < 0.001, did not reach statistical significance.

#### 3.1.2. Mean Performance

**Figure 2:**
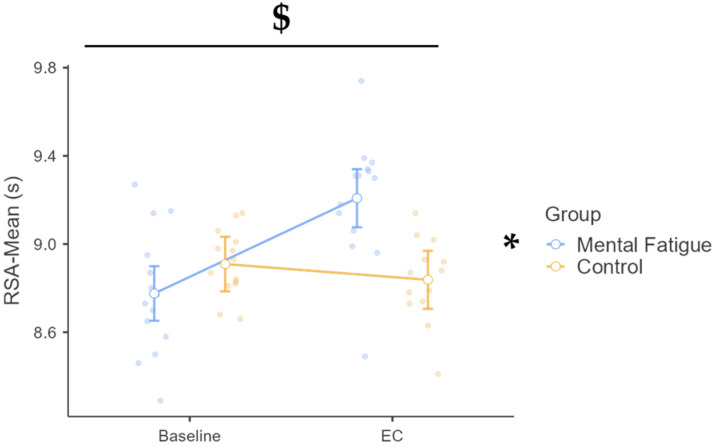
Repeated sprint ability (RSA): mean sprint time across phases and groups. Note: RSA-Mean Sprint Time (mean ± SE) for the mental fatigue and control groups across sessions (pre vs. post). $ = significant group × session interaction; * = significant main effect of session (Time). RSA-Mean Sprint Time: A significant group × session interaction ($ *p* < 0.05) and a significant main effect of session (* *p* < 0.05) were identified.

A significant time × group interaction was observed, F(1, 26) = 21.73, *p* < 0.001, η^2^_p_ = 0.455, 90% CI [0.20, 0.61], with the RSA-Mean increasing from baseline to EC in the MF group but not in the CON group (Figure 2). The MF group showed a large increase (Δ = 0.432 s, *p* < 0.001, d = 1.86, 95% CI [0.67, 3.05]), while the CON group showed no change (Δ = 0.071 s, *p* = 1.000, d = 0.31, 95% CI [−0.64, 1.25]). At EC, MF was significantly slower than CON (Δ = 0.370 s, *p* = 0.002, d = 1.59, 95% CI [0.31, 2.87]). A main effect of time was also significant, F(1, 26) = 11.15, *p* = 0.003, η^2^_p_ = 0.300, 90% CI [0.07, 0.48], but no baseline group difference was found, F(1, 26) = 2.91, *p* = 0.100, η^2^_p_ = 0.101.

#### 3.1.3. Total Time

**Figure 3:**
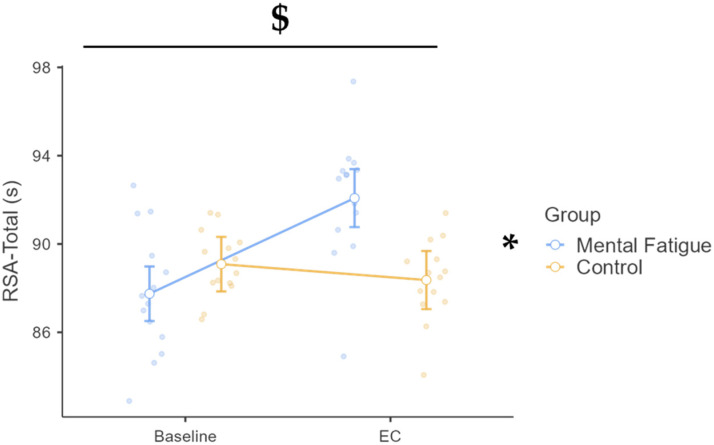
Repeated sprint ability (RSA): total time across phases and groups. Note: RSA-Total Time (mean ± SE) for the mental fatigue and control groups across sessions (pre vs. post). Symbols denote statistical significance: $ = group × session interaction (*p* < 0.05); * = main effect of session (time), (*p* < 0.05).

A significant time × group interaction was found, F(1, 26) = 22.09, *p* < 0.001, η^2^_p_ = 0.459, 90% CI [0.21, 0.61], with total sprint time increasing from baseline to the EC in the MF group but not in the CON group (Figure 3). The MF group showed a large increase (Δ = 4.331 s, *p* < 0.001, d = 1.87, 95% CI [0.67, 3.06]), whereas CON showed no change (Δ = 0.721 s, *p* = 1.000, d = 0.31, 95% CI [−0.63, 1.25]). At EC, MF was significantly slower than CON (Δ = 3.711 s, *p* = 0.002, d = 1.60, 95% CI [0.32, 2.88]). A main effect of time was also observed, F(1, 26) = 11.27, *p* = 0.002, η^2^_p_ = 0.302, 90% CI [0.08, 0.31], but groups did not differ at baseline, F(1, 26) = 2.92, *p* = 0.099, η^2^_p_ = 0.101.

#### 3.1.4. CV and RSA Performance Decrement (Dec%)

Although the time × group interaction did not reach significance, F(1, 26) = 3.65, *p* = 0.067, η^2^_p_ = 0.123, a significant main effect of time was found, F(1, 26) = 14.26, *p* < 0.001, η^2^_p_ = 0.354, 90% CI [0.11, 0.53], with CV increasing from baseline to EC. Post hoc comparisons revealed a significant rise in the MF group (Δ = 1.656%, *p* = 0.003, d = 1.19, 95% CI [−2.15, −0.22]), whereas no corresponding change was evident in the CON group (Δ = 0.544%, *p* = 1.000, d = 0.39). No significant group differences were found at baseline, F(1, 26) = 1.86, *p* = 0.184, η^2^_p_ = 0.067.

The analysis for RSA Dec% revealed a significant main effect of time, F(1, 26) = 10.55, *p* = 0.003, η^2^_p_ = 0.289, 90% CI [0.07, 0.47], indicating that Dec% significantly increased from baseline to post-intervention across both groups. However, there was no significant time × group interaction, F(1, 26) = 0.47, *p* = 0.499, η^2^_p_ = 0.018, suggesting that the change in Dec% over time was not statistically different between the MF and CON groups. The main effect of the group was also not significant, F(1, 26) = 1.06, *p* = 0.313, η^2^_p_ = 0.039. Post hoc comparisons (Bonferroni-adjusted) revealed no statistically significant differences between groups at any time point (*p* > 0.05). Notably, the largest mean difference occurred between the MF group at baseline and after the EC (Δ = −3.40, *p* = 0.060, d = −0.85, 95% CI [−6.88, 0.09]), reflecting a non-significant increased fatigue-induced performance decrement. Similarly, the contrast between the CON and MF groups post-intervention approached significance (Δ = −4.07, *p* = 0.071, d = −1.02), indicating a moderate effect size, despite non-significance after correction.

### 3.2. Performance Measures—Countermovement Jump (CMJ)

#### Height

No significant time × group interaction was found for CMJ height, F(1, 26) = 0.13, *p* = 0.722, η^2^_p_ = 0.005, nor was there a main effect of time, F(1, 26) = 0.01, *p* = 0.906, η^2^_p_ < 0.001. The group effect was also not significant, F(1, 26) = 0.86, *p* = 0.361, η^2^_p_ = 0.032. Post hoc comparisons showed no meaningful changes from baseline to EC in either group (all *p* > 0.95, all d < 0.40), indicating that CMJ performance remained unchanged.

### 3.3. Performance Measures—Repeated Jumping Ability (RJA)

#### 3.3.1. Average Height

**Figure 4:**
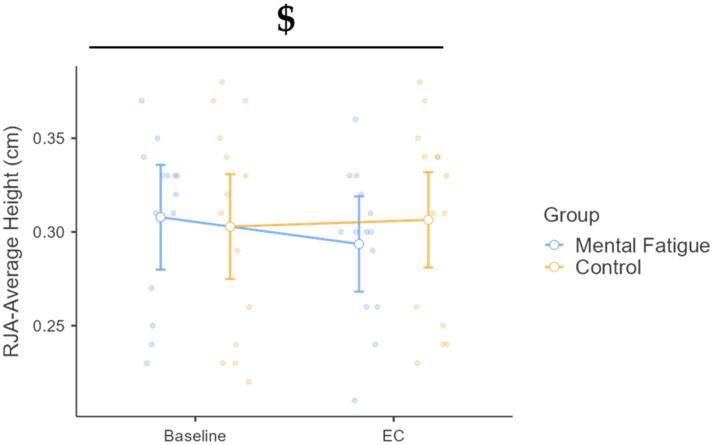
Repeated jump ability (RJA): average jump height across phases and groups. Note: RJA-Average Jump Height (mean ± SE) for the mental fatigue and control groups across sessions (pre vs. post). $ = significant group × session interaction. RJA-Average Jump Height: a significant group × session interaction ($ *p* < 0.05) was identified.

A significant time × group interaction was found, F(1, 26) = 8.71, *p* = 0.007, η^2^_p_ = 0.251, 90% CI [0.05, 0.44], indicating different trajectories between groups from baseline to EC (Figure 4). Although the main effect of time was not significant, F(1, 26) = 3.14, *p* = 0.088, η^2^_p_ = 0.108, post hoc comparisons revealed a small but significant increase in the MF group (Δ = 0.014 m, *p* = 0.015, d = 0.29, 95% CI [0.002, 0.027]), with no change in the CON group (Δ = −0.004 m, *p* = 1.000, d = −0.07, 95% CI [−0.33, 0.18]). No between-group difference was observed at EC (Δ = 0.013 m, *p* = 1.000, d = 0.26, 95% CI [−0.77, 1.30]). The main effect of the group was also not significant, F(1, 26) = 0.05, *p* = 0.830, η^2^_p_ = 0.002.

#### 3.3.2. Contact Time

**Figure 5:**
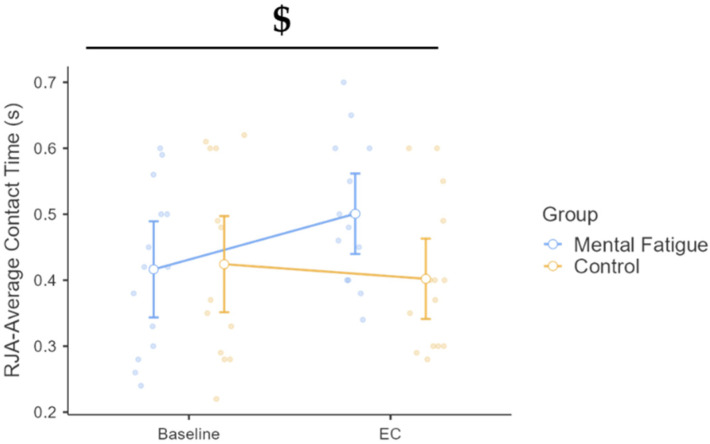
Repeated jump ability (RJA): average contact time across phases and groups. Note: RJA-Average Contact Time (mean ± SE) for the mental fatigue and control groups across sessions (pre vs. post). $ = significant group × session interaction. RJA-Average Contact Time: a significant group × session interaction ($ *p* < 0.05) was identified.

A significant time × group interaction was found, F(1, 26) = 10.47, *p* = 0.003, η^2^_p_ = 0.287, 90% CI [0.07, 0.47], indicating divergent responses between groups from baseline to the EC (Figure 5). Although the main effect of time did not reach significance, F(1, 26) = 3.57, *p* = 0.070, η^2^_p_ = 0.121, post hoc analysis revealed a significant increase in contact time for the MF group (Δ = 0.084 s, *p* = 0.007, d = 0.69, 95% CI [0.018, 0.151]), whereas the CON group showed no significant change (Δ = 0.022 s, *p* = 1.000, d = 0.18, 95% CI [−0.37, 0.73]). No significant difference was found between groups at EC (Δ = −0.099 s, *p* = 0.159, d = −0.81, 95% CI [−1.84, 0.22]). The main effect of the group was also non-significant, F(1, 26) = 1.10, *p* = 0.303, η^2^_p_ = 0.041.

### 3.4. Cognitive and Subjective Measures—PVT

#### 3.4.1. Reaction Time

**Figure 6:**
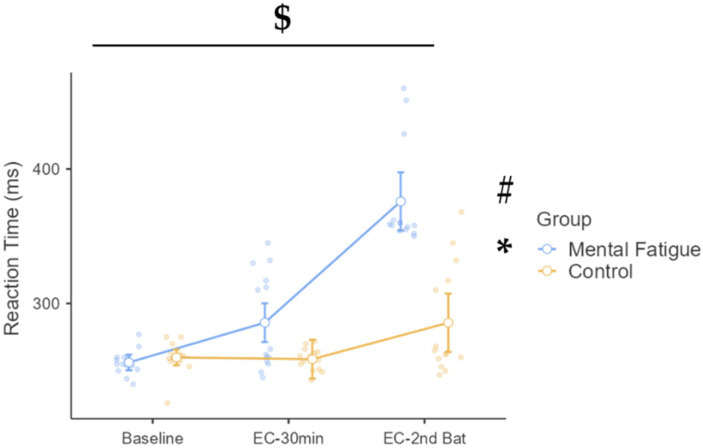
Psychomotor vigilance task: reaction time (RT) across phases and groups. Note: RT (mean ± SE) of reaction time (RT, in milliseconds) measured using the psychomotor vigilance task (PVT) for the mental fatigue and control groups across three time points: baseline, EC–30 min (after 30 min of the executive control task), and EC–2nd Battery (after completion of the second battery of physical/cognitive tasks). $ = significant group × session interaction; * = significant main effect of session (time); # = significant main effect of group (condition). RT: a significant group × session interaction ($ *p* < 0.05), a significant main effect of session (* *p* < 0.05), and a significant main effect of group (# *p* < 0.05) were identified.

A 2 (group: CON vs. MF) × 3 (time: baseline, EC–30 min, EC–2nd Battery) mixed ANOVA was conducted to examine changes in reaction time (RT) and the number of lapses over time between groups (Figure 6). Mauchly’s test indicated that the assumption of sphericity was violated for the within-subjects factor, χ^2^(2) = 12.06, *p* = 0.002; therefore, Greenhouse–Geisser corrections were applied to the degrees of freedom in all relevant analyses. A significant time × group interaction was observed, F(1.45, 37.61) = 20.71, *p* < 0.001, η^2^_p_ = 0.443, 90% CI [0.23, 0.57], indicating differential changes in reaction time across conditions between the MF and CON groups. The main effect of time was also significant, F(1.45, 37.61) = 53.77, *p* < 0.001, η^2^_p_ = 0.674, 90% CI [0.51, 0.76], reflecting a general increase in RT across sessions. Additionally, a significant main effect of group emerged, F(1, 26) = 37.58, *p* < 0.001, η^2^_p_ = 0.591, 90% CI [0.36, 0.71], with the mental fatigue group exhibiting slower RTs overall.

Post hoc comparisons revealed a large and significant increase in RT within the MF group from baseline to EC–2nd Battery (Δ = 119.71 ms, *p* < 0.001, d = 4.15, 95% CI [85.41, 154.02]). In contrast, the CON group did not exhibit a statistically significant change (Δ = 25.79 ms, *p* = 0.335, d = 0.92, 95% CI [−8.52, 60.09]). At EC–2nd Battery, RTs in the MF group were significantly slower than those in the CON group (Δ = 90.21 ms, *p* < 0.001, d = 3.22, 95% CI [42.07, 138.36]).

#### 3.4.2. Lapses

**Figure 7:**
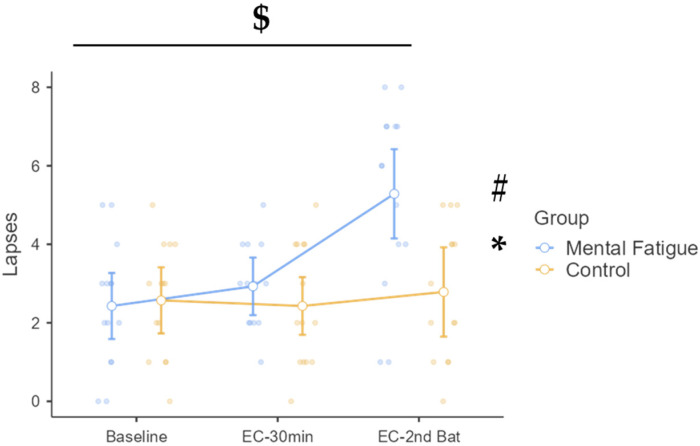
Psychomotor vigilance task: number of lapses across phases and groups. Note: Lapses (mean ± SE) recorded in the psychomotor vigilance task (PVT) for the mental fatigue and control groups across three time points: baseline, EC–30 min (after 30 min of the executive control task), and EC–2nd Battery (after completion of the second battery of physical/cognitive tasks). $ = significant group × session interaction; * = significant main effect of session (time); # = significant main effect of group (condition). Lapses: A significant group × session interaction ($ *p* < 0.05), a significant main effect of session (* *p* < 0.05), and a significant main effect of group (# *p* < 0.05) were identified.

Mauchly’s test indicated that the assumption of sphericity was not violated for the within-subjects factor, χ^2^(2) = 1.69, *p* = 0.429; therefore, sphericity was assumed in all analyses. A significant time × group interaction was found, F(1.88, 48.81) = 8.51, *p* < 0.001, η^2^_p_ = 0.247, 90% CI [0.08, 0.38], indicating differential patterns of lapses over time between the CON and MF groups (Figure 7). The main effect of time was also significant, F(1.88, 48.81) = 12.64, *p* < 0.001, η^2^_p_ = 0.327, 90% CI [0.14, 0.46], suggesting that the number of lapses varied across the three measurement points. The main effect of the group was not statistically significant, F(1, 26) = 3.61, *p* = 0.069, η^2^_p_ = 0.122, although the MF group exhibited a non-significantly higher number of lapses compared to the CON group.

Post hoc comparisons showed that the MF group exhibited a significant increase in lapses from baseline to EC–2nd Battery (Δ = 2.86 lapses, *p* < 0.001, d = 1.71, 95% CI [1.20, 4.52]) and from EC–30 min to EC–2nd Battery (Δ = 2.36 lapses, *p* < 0.001, d = 1.41, 95% CI [0.78, 3.93]). At EC–2nd Battery, the number of lapses in the MF group was significantly higher than in the CON group (Δ = 2.50 lapses, *p* = 0.054, d = 1.49), though this comparison narrowly missed Bonferroni-corrected significance. These results support the negative impact of mental fatigue on sustained attention, particularly at the later stage of testing.

### 3.5. Cognitive and Subjective Measures—Ratings of Perceived Exertion (RPE)

**Figure 8:**
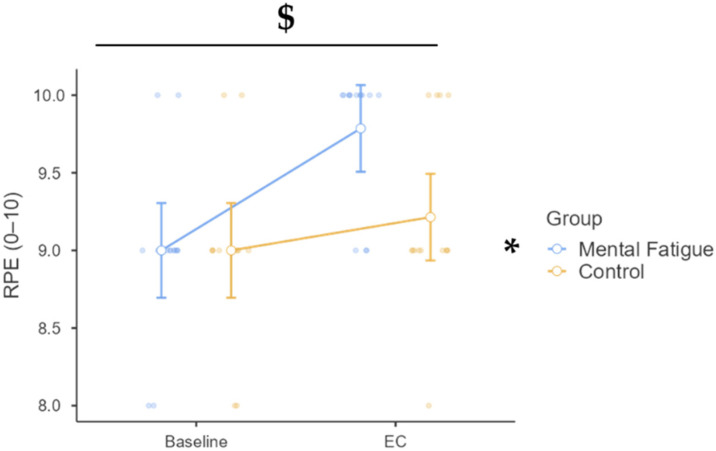
Ratings of perceived exertion (RPE) across phases and groups. Note: RPE (mean ± SE) for the mental fatigue (MF) and control (CON) groups across sessions (pre vs. post). $ = significant group × session interaction; * = significant main effect of session (time). RPE: A significant group × session interaction ($ *p* < 0.05) and a significant main effect of session (* *p* < 0.05) were identified.

Regarding perceived exertion across time and between groups (Figure 8), a significant time × group interaction was found, F(1, 26) = 4.67, *p* = 0.040, η^2^_p_ = 0.152, 90% CI [0.00, 0.35], indicating differential changes in RPE between groups from baseline to the EC. The main effect of time was also significant, F(1, 26) = 14.32, *p* < 0.001, η^2^_p_ = 0.355, 90% CI [0.11, 0.53], suggesting that RPE increased overall from baseline to EC. The main effect of group did not reach statistical significance, F(1, 26) = 3.56, *p* = 0.071, η^2^_p_ = 0.120.

Post hoc comparisons revealed that the MF group experienced a significant increase in RPE from baseline to EC (Δ = 0.79, *p* = 0.002, d = 1.48, 95% CI [0.25, 1.32]), while the CON group did not (Δ = 0.21, *p* = 1.000, d = 0.40, 95% CI [−0.32, 0.75]). At the EC time point, participants in the MF group reported significantly higher RPE than those in the CON group (Δ = 0.57, *p* = 0.038, d = 1.07, 95% CI [0.02, 1.12]). These results suggest that mental fatigue exacerbated the subjective perception of effort during the experimental condition.

### 3.6. Cognitive and Subjective Measures—NASA TLX

#### 3.6.1. Mental Demand

A 2 (group: MF vs. CON) × 3 (phase: baseline, EC-30 min, EC-2nd Battery) mixed ANOVA was conducted to evaluate differences in self-reported mental demand across testing phases (Table 1). Mauchly’s test of sphericity was non-significant, χ^2^(2) = 4.92, *p* = 0.085, indicating that the assumption of sphericity was met. Therefore, Greenhouse–Geisser corrections were applied. A significant time × group interaction was observed, F(1.697, 44.12) = 37.96, *p* < 0.001, η^2^_p_ = 0.593, 90% CI [0.42, 0.69], indicating differential changes in mental demand across phases between groups. The main effect of phase was also significant, F(1.697, 44.12) = 48.19, *p* < 0.001, η^2^_p_ = 0.650, 90% CI [0.49, 0.73], reflecting overall increases in perceived mental demand. Furthermore, a significant main effect of the group emerged, F(1, 26) = 117.93, *p* < 0.001, η^2^_p_ = 0.819, 90% CI [0.69, 0.87], with the MF group consistently reporting higher mental demand than the CON group.

Post hoc analyses (Bonferroni-adjusted) revealed that the MF group experienced a substantial increase in mental demand from baseline to EC-30 min (Δ = 40.00, *p* < 0.001, d = 3.565, 95% CI [−55.07, −24.93]) and from baseline to EC-2nd Battery (Δ = 49.29, *p* < 0.001, d = 4.392, 95% CI [−63.59, −34.99]). In contrast, the CON group exhibited no significant changes across phases. Between-group comparisons at EC-30 min and EC-2nd Battery showed that the MF group reported significantly greater mental demand than the CON group (Δ = 45.00 and Δ = 40.00, respectively, both *p* < 0.001), supporting the effectiveness of the experimental manipulation in elevating perceived mental workload.

#### 3.6.2. Physical Demand

For physical demand across testing phases, Mauchly’s test indicated a violation of the sphericity assumption, χ^2^(2) = 6.90, *p* = 0.032; therefore, Greenhouse–Geisser corrections were applied (ε = 0.806). The main effect of the phase was significant, F(1.611, 41.90) = 1182.08, *p* < 0.001, η^2^_p_ = 0.978, indicating substantial changes in perceived physical demand across the three time points. However, neither the time × group interaction (F(1.611, 41.90) = 0.95, *p* = 0.378, η^2^_p_ = 0.035) nor the main effect of group (F(1, 26) = 0.02, *p* = 0.888, η^2^_p_ < 0.001) reached statistical significance, suggesting that physical demand patterns did not differ significantly between the groups.

Post hoc analyses (Bonferroni-adjusted) revealed that both groups experienced significant increases in physical demand from baseline to EC-30 min. Specifically, for the control group, the increase was substantial (d = 10.64, 95% CI [5.716, 15.563]), and a comparable increase was observed in the mental fatigue group (d = 10.34, 95% CI [5.546, 15.131]). These elevations in perceived physical demand were followed by significant reductions from EC-30 min to EC-2nd Battery, with d = −10.84, 95% CI [−15.772, −5.908] for the control group and d = −11.24, 95% CI [−16.351, −6.133] for the mental fatigue group. No significant between-group differences were observed at any time point (all *p* > 0.632), indicating that physical demand increased sharply following the experimental manipulation and then decreased similarly in both groups during the second battery, irrespective of mental fatigue condition.

#### 3.6.3. Effort

Regarding effort across testing phases, Mauchly’s test indicated a violation of the sphericity assumption, χ^2^(2) = 12.79, *p* = 0.002; thus, Greenhouse–Geisser corrections were applied (ε = 0.714). The analysis revealed a significant main effect of the phase, F(1.43, 37.13) = 270.02, *p* < 0.001, η^2^_p_ = 0.912, 90% CI [0.86, 0.93], as well as a significant phase × group interaction, F(1.43, 37.13) = 16.03, *p* < 0.001, η^2^_p_ = 0.381, 90% CI [0.17, 0.52], indicating that changes in effort differed between the two groups. Additionally, the main effect of the group was significant, F(1, 26) = 36.38, *p* < 0.001, η^2^_p_ = 0.583, 90% CI [0.35, 0.70].

Post hoc pairwise comparisons (Bonferroni-adjusted) showed that both groups experienced significant increases in effort from baseline to EC-30 min; for the control group, d = 6.39, 95% CI [3.119, 9.656]; for the mental fatigue group, d = 3.47, 95% CI [1.374, 5.565]. Notably, the MF group reported significantly higher effort than the CON group at EC-30 min (Δ = 26.43, *p* < 0.001, d = −2.92, 95% CI [−4.565, −1.270]). From EC-30 min to EC-2nd Battery, both groups showed significant reductions in effort, with d = −7.96, 95% CI [−11.626, −4.303] for the control group and d = −8.12, 95% CI [−11.865, −4.379] for the MF group. Furthermore, the MF group reported significantly greater effort than the control group at both EC-30 min and EC-2nd Battery (all *p* < 0.001), whereas no significant difference was observed at baseline (*p* = 1.000), indicating that the mental fatigue manipulation successfully elevated perceived effort during task execution and sustained this effect through the second battery.

#### 3.6.4. Frustration

Similarly, a 2 (group: MF vs. CON) × 3 (phase: baseline, EC-30 min, EC-2nd Battery) mixed ANOVA was conducted to examine changes in perceived frustration. Mauchly’s test confirmed that the assumption of sphericity was met, χ^2^(2) = 1.61, *p* = 0.447; therefore, sphericity was assumed. The analysis revealed a significant main effect of phase, F(1.88, 48.95) = 8.61, *p* < 0.001, η^2^_p_ = 0.249, indicating overall differences in frustration levels across the three phases. However, no significant interaction between phases and groups was observed, F(1.88, 48.95) = 0.46, *p* = 0.625, η^2^_p_ = 0.017, suggesting that the pattern of change over time did not differ between groups. The main effect of the group was also non-significant, F(1, 26) = 1.46, *p* = 0.237, η^2^_p_ = 0.053.

Post hoc comparisons (Bonferroni-adjusted) showed that frustration decreased significantly from EC-30 min to EC-2nd Battery in the mental fatigue group (*p* = 0.012, d = −1.43, 95% CI [−2.810, −0.052]). No other pairwise comparisons reached significance, indicating that while perceived frustration fluctuated over time, these changes were not differentially influenced by mental fatigue manipulation between groups.

### 3.7. Cognitive and Subjective Measures—M-Vas

Initially, a 2 (group: CON vs. MF) × 2 (time: baseline vs. EC–30 min) mixed ANOVA was conducted to assess the persistence of subjective mental fatigue over time. A significant time × group interaction was observed, F(1, 26) = 116.03, *p* < 0.001, η^2^_p_ = 0.817, 90% CI [0.68, 0.87], indicating that changes in fatigue levels from baseline to 30 min post-experimental condition (EC–30 min) differed significantly between groups. The main effect of time was also significant, F(1, 26) = 617.09, *p* < 0.001, η^2^_p_ = 0.960, 90% CI [0.93, 0.97], demonstrating an overall increase in fatigue over time. In addition, a significant main effect of the group was found, F(1, 26) = 84.59, *p* < 0.001, η^2^_p_ = 0.765, 90% CI [0.60, 0.83], confirming that the MF group reported higher fatigue levels than the CON group across time points.

Post hoc comparisons revealed a significant increase in M-VAS scores from baseline to EC–30 min in the MF group (Δ = 5.79, *p* < 0.001, d = 8.74, 95% CI [5.13, 6.44]), while the CON group also exhibited a statistically significant but smaller increase (Δ = 2.29, *p* < 0.001, d = 3.45, 95% CI [1.63, 2.94]). Furthermore, at EC–30 min, the MF group reported significantly higher fatigue levels than the CON group (Δ = 3.50, *p* < 0.001, d = 5.29, 95% CI [2.53, 4.47]).

**Figure 9:**
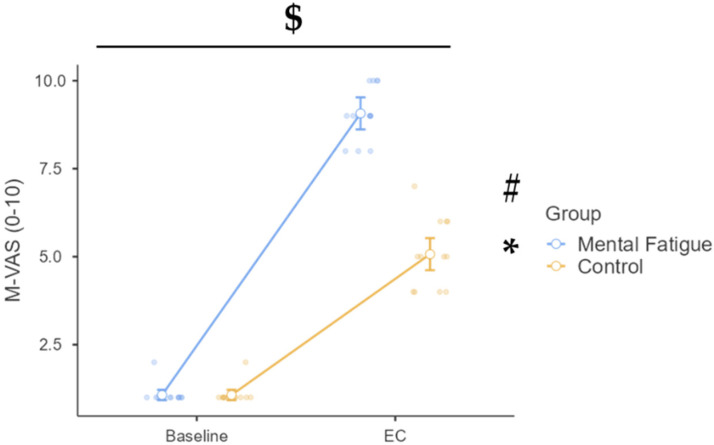
Mental Visual Analog Scale (M-VAS) across phases (baseline and EC) and groups. Note: M-VAS (mean ± SE) for the mental fatigue and control groups across sessions, baseline and EC (after completion of the second battery of physical/cognitive tasks). $ = significant group × session interaction; * = significant main effect of session (time); # = significant main effect of group (condition). M-VAS: a significant group × session interaction ($ *p* < 0.05), a significant main effect of session (* *p* < 0.05), and a significant main effect of group (# *p* < 0.05) were identified.

Subsequently, a 2 (group: control vs. mental fatigue) × 2 (time: baseline vs. EC) mixed ANOVA was conducted to assess changes in self-reported mental fatigue (Figure 9). A significant time × group interaction emerged, F(1, 26) = 132.36, *p* < 0.001, η^2^_p_ = 0.836, 90% CI [0.71, 0.88], indicating different patterns of change between the two groups from baseline to the experimental condition (EC). The main effect of time was also significant, F(1, 26) = 1191.27, *p* < 0.001, η^2^_p_ = 0.979, 90% CI [0.96, 0.98], demonstrating an overall increase in mental fatigue. Additionally, a significant main effect of the group was found, F(1, 26) = 167.08, *p* < 0.001, η^2^_p_ = 0.865, 90% CI [0.76, 0.90], suggesting that the mental fatigue group reported higher fatigue scores overall.

Post hoc analysis confirmed a substantial increase in M-VAS scores for the mental fatigue group from baseline to EC (Δ = 8.00, *p* < 0.001, d = 12.99, 95% CI [7.30, 8.70]), compared to a smaller yet statistically significant increase in the CON group (Δ = 4.00, *p* < 0.001, d = 6.50, 95% CI [3.30, 4.70]). Furthermore, at the EC time point, the mental fatigue group reported significantly greater fatigue than the control group (Δ = 4.00, *p* < 0.001, d = 6.50, 95% CI [3.11, 4.89]). These findings clearly demonstrate that mental fatigue manipulation was effective, resulting in a considerably higher subjective experience of fatigue in the experimental group.

### 3.8. Cognitive and Subjective Measures—Motivation

A 2 (group) × 2 (time) mixed ANOVA revealed no significant main effects or interaction for self-reported motivation, all ps > 0.32. Specifically, the time × group interaction was non-significant, F(1, 26) = 1.00, *p* = 0.327, η^2^_p_ = 0.037, suggesting that the experimental manipulation did not influence participants’ motivation levels.

## 4. Discussion

The present study investigated the influence of mental fatigue (MF), induced by a 30 min Stroop task, on repeated sprint ability (RSA), repeated jump ability (RJA), psychomotor vigilance, and perceived exertion in trained individuals. In line with our hypotheses and replicating prior findings [9], MF negatively affected mean RSA and RJA performance while leaving peak performance in singular tasks unaffected. Furthermore, MF led to a significant deterioration in sustained attention and vigilance, as evidenced by slower reaction times and increased lapses in the psychomotor vigilance task (PVT), and was associated with greater perceived exertion (RPE). These findings reinforce the assertion that MF impairs performance when cognitive and neuromuscular demands are combined, particularly in strength endurance contexts requiring repeated effort and cognitive engagement. This aligns with recent work demonstrating that MF selectively impairs neuromuscular performance and increases perceived effort [6].

Regarding RSA performance, MF impaired mean sprint times and increased total sprint duration while exerting no influence on best sprint time. This distinction reinforces the idea that MF selectively compromises performance across tasks requiring repeated bursts of effort rather than singular maximal outputs. These results are congruent with prior work showing that while MF does not degrade the maximal speed achieved in a single sprint [10], it reduces the consistency and overall quality of performance during repeated efforts, likely due to increased perceived exertion and reduced attentional resources needed to maintain motor control and pacing over time [9,57]. It should be acknowledged, however, that the RSA decrement only approached statistical significance and was accompanied by modest effect sizes, warranting cautious interpretation regarding its practical relevance. The increased total time and degraded mean performance observed in our MF group support the interpretation that MF interferes with neuromotor regulation and pacing strategies during intermittent high-intensity activities involving directional changes.

Similarly, RJA performance was impaired under MF. Participants in the MF condition showed a significant decline in average jump height and an increase in ground contact time, yet their performance on the maximal CMJ remained stable. This dissociation aligns with earlier findings suggesting that MF has minimal or no effect on isolated maximal power output [58,59], but it can compromise strength endurance and neuromuscular coordination during repeated efforts [9,60]. The increased contact time observed in the MF group may reflect compromised postural control or reactive stabilization, potentially linked to diminished central nervous system engagement due to cognitive overload. Although biomechanical parameters were not directly assessed in the current study, these changes suggest that MF may impair the fine motor adjustments required during rapid eccentric–concentric cycles typical of repeated jumping.

In addition to impairing physical performance, MF also disrupted cognitive functioning. Reaction times during the PVT were significantly slower in the MF group after both the Stroop task and the physical test battery, with a notable increase in attentional lapses. This pattern reflects an additive effect of cognitive and physical fatigue on sustained attention, mirroring prior research [9,45]. Comparable impairments in psychomotor vigilance and reaction time under MF have been reported, with large effect sizes indicating compromised attentional control and increased response latency [6]. The observed cognitive impairments are consistent with models positing that MF undermines top–down attentional control and increases response variability [1,2]. These deficits are particularly concerning in athletic contexts where split-second decisions and accurate perception–action coupling are critical for optimal performance.

Perceived effort, as indexed by RPE, was also significantly elevated following the MF intervention, despite no differences in physiological demand across groups. This finding supports the psychobiological model of fatigue, which emphasizes that subjective effort mediates performance in endurance and repeated-effort tasks [61]. As in previous work, the heightened RPE in the MF group suggests that the central perception of task difficulty is modulated by cognitive state, independent of metabolic or cardiovascular strain. Our findings corroborate the idea that the brain integrates both cognitive and physiological signals to gauge effort, which in turn influences motivation and pacing [12]. Interestingly, despite these increases in perceived exertion, motivation levels did not differ between groups. This finding aligns with previous studies suggesting that MF alters subjective effort without necessarily reducing task motivation [6,9]. It is possible that the trained status of our participants buffered motivational declines, allowing them to sustain commitment to the task even under greater perceived effort. Nonetheless, the absence of motivational changes should be interpreted cautiously, as self-report measures may not fully capture subtle fluctuations in intrinsic drive.

The present study builds on recent evidence that MF specifically impairs strength endurance and complex neuromotor tasks, rather than maximal outputs. From a practical standpoint, these results suggest that MF may have consequences in sports contexts that require frequent, intense, and precisely coordinated actions under mentally demanding conditions. Athletes and coaches should consider MF as a modifiable constraint that can compromise performance, particularly in the latter stages of games or matches when cognitive and physical fatigue co-occur.

Nevertheless, this study is not without limitations. First, we did not include biomechanical assessments, which would have allowed for a more granular analysis of neuromuscular control during jumping and sprinting. Future studies should incorporate motion analysis or surface electromyography to capture postural sway, inter-limb coordination, or muscular activation patterns under fatigue. Second, our participant sample consisted of trained amateur athletes; it remains unclear whether elite performers would demonstrate similar or reduced susceptibility to MF due to superior cognitive resilience or neurophysiological conditioning. Comparative investigations across skill levels are warranted to address this issue. Lastly, while we used a well-established Stroop task to induce MF, the ecological validity of this induction protocol in real-world sport scenarios may be limited. Future research should explore more ecologically relevant mental loads, such as tactical decision making tasks or video-based anticipation exercises, to better replicate the cognitive challenges faced in competitive sport.

In summary, our findings support and extend prior work demonstrating that MF impairs repeated, high-intensity physical performance and cognitive vigilance. MF did not alter peak sprint or jump performance, but it degraded strength endurance and heightened perceived exertion. MF also worsened vigilance (slower reaction times and more lapses), heightened perceived exertion, and increased mental demand and effort without affecting motivation, aligning with evidence that MF impairs sport-specific psychomotor/motor performance via attentional and control limitations [62,63,64] and reduces technical accuracy under concurrent cognitive load [65], with central processing costs reflected in prolonged reaction time [66]. Coaches and practitioners should recognize MF as a performance-limiting factor and consider strategies to monitor and mitigate its effects. Interventions may include psychological techniques and training, such as brain endurance training [67] to enhance attentional control and reduce effort and MF and environmental, nutritional, and recovery modalities aimed at reducing cognitive load [68,69,70]. Moreover, embedding structured cognitive challenges within physical training may build resilience to mental fatigue (MF) and preserve performance under pressure, in line with contemporary theoretical accounts of motivational control and resource depletion [71].

## 5. Conclusions

In summary, MF, induced by prolonged cognitive demand, impairs repeated direction sprinting and jumping, elevates perceived effort, and degrades sustained attention without affecting single maximal outputs or physiological markers. These effects are consistent across sports and task types. Sports scientists and practitioners should monitor cognitive fatigue and integrate resilience building interventions to preserve performance during mentally and physically demanding scenarios.

## Figures and Tables

**Table 1:** NASA-TLX scores: means and standard deviations across phases for mental fatigue and control groups.

Variable	Group	Baseline Mean (SD)	EC-30′ Mean (SD)	EC-2nd Bat. Mean (SD)
**Mental Demand**	MF	32.9 (14.9)	72.9 (7.26)	82.1 (9.75)
	CON	34.3 (16.04)	27.9 (5.79)	42.1 (9.75)
**Physical Demand**	MF	83.6 (11.51)	10.0 (0.01)	90.0 (6.79)
	CON	85.7 (8.52)	10.0 (0.01)	87.1 (7.26)
**Effort**	MF	71.4 (11.67)	40.7 (8.29)	87.9 (5.79)
	CON	72.1 (13.69)	14.3 (6.46)	86.4 (4.97)
**Frustration**	MF	28.6 (10.27)	28.6 (9.49)	40.0 (12.40)
	CON	28.6 (11.67)	24.3 (10.89)	35.0 (10.92)

Note: Values are expressed as mean (standard deviation). MF = mental fatigue group; CON = control group; EC–30′ = after 30 min of the executive control task; EC–2nd Bat. = after completion of the second battery of tasks. NASA-TLX = National Aeronautics and Space Administration Task Load Index. Subscales include mental demand, physical demand, effort, and frustration.

## Data Availability

The original contributions presented in the study are included in the article, further inquiries can be directed to the corresponding author/s.
